# The pyroptosis-related gene signature predicts prognosis and indicates immune activity in hepatocellular carcinoma

**DOI:** 10.1186/s10020-022-00445-0

**Published:** 2022-02-05

**Authors:** Min Deng, Shiquan Sun, Rongce Zhao, Renguo Guan, Zhen Zhang, Shaohua Li, Wei Wei, Rongping Guo

**Affiliations:** 1 Department of Liver Surgery, Sun Yat-sen University Cancer Center, 651 Dongfeng East Road, Guangzhou, 510060 China; 2 State Key Laboratory of Oncology in South China, Collaborative Innovation Center for Cancer Medicine, Guangzhou, 510060 China; 3 Research Unit of Precision Diagnosis and Treatment for Gastrointestinal Cancer, Chinese Academy of Medical Sciences, Guangzhou, 510060 China; 4 Department of Dermatovenereology, The Seventh Affiliated Hospital, Sun Yat-sen University, 518000 Shenzhen, China

**Keywords:** Hepatocellular carcinoma, Pyroptosis, Prognosis, Immunity, Nomogram

## Abstract

**Background:**

Hepatocellular carcinoma (HCC) remains one of the most common malignant tumors with poor survival. Pyroptosis is a kind of programmed cell death that can regulate the proliferation, invasion, and metastasis of tumor cells. However, the expression levels of pyroptosis-related genes (PRGs) in HCC and their relationship with prognosis are still unclear.

**Methods:**

Our study identified 35 PRGs through bioinformatics analysis that were differentially expressed between tumor samples and nontumor samples. According to these differentially expressed genes, HCC patients could be divided into two groups, cluster 1 and cluster 2. The least absolute shrinkage and selection operator (LASSO) Cox regression method was performed to construct a 10-gene signature that classified HCC patients in the cancer genome atlas (TCGA) database into low-risk and high-risk groups.

**Results:**

The results showed that the survival rate of HCC patients in the low-risk group was significantly higher than that in the high-risk group (p < 0.001). The validation cohort, the Gene Expression Omnibus (GEO) cohort, was divided into two risk groups based on the median risk score calculated by the TCGA cohort. The overall survival (OS) of the low-risk group was significantly better than that of the high-risk group (p = 0.007). Univariate and multivariate Cox regression analyses revealed that the risk score was an independent factor in predicting OS in HCC patients. Gene Ontology and Kyoto Encyclopedia of Genes and Genomes analyses showed that immune-related high-risk groups were rich in genes and had reduced immune status.

**Conclusions:**

PRGs play a significant role in tumor immunity and have the potential capability to predict the prognosis of HCC patients.

**Supplementary Information:**

The online version contains supplementary material available at 10.1186/s10020-022-00445-0.

## Background

Hepatocellular carcinoma (HCC) is the most common primary liver cancer, and it is also the fourth most common malignant tumor, with high mortality and a high degree of malignancy in humans (Villanueva 2019). 80% of patients are in the advanced stage at the first visit and lose the opportunity for radical surgery (Forner et al. 2018; Vibert et al. 2020). Although some HCC patients undergo radical hepatectomy, a high rate of postoperative recurrence and metastasis are still major challenges for the survival of patients (Anwanwan et al. 2020). In recent years, targeted therapy and immune checkpoint inhibitor (ICI) therapy have made significant progress in various malignancies and achieved satisfactory efficacy in HCC (Greten et al. 2019; Llovet et al. 2018). However, there are still a considerable proportion of individuals with poor survival (Chen et al. 2019; Cheng et al. 2020). Hence, it is necessary to explore new targets to improve the therapeutic efficacy of HCC.

Pyroptosis, also recognized as inflammatory necrosis, is a new type of programmed cell death (Kovacs and Miao 2017), which has been proven to be closely related to the inflammatory response, sepsis, and tumor chemotherapy (Frank and Vince 2019). Significant findings suggest that the occurrence of pyroptosis is closely associated with tumor immunity and can predict and improve the efficacy of immunotherapy (Tang et al. 2020; Orning et al. 2019). In recent years, the systemic treatment of ICIs based on programmed cell death protein 1 (PD-1)/programmed cell death receptor ligand 1 (PD-L1) combined with targeted drugs and various local therapies has made noteworthy progress in advanced HCC patients (Anwanwan et al. 2020; Greten et al. 2019). Therefore, it is of great importance for the treatment of HCC, especially immunotherapy, to identify pyroptosis-related genes (PRGs) and analyze their roles and relationship with immunity.

The gasdermin family is the main executor of pyroptosis and includes gasdermin-A (GSDMA), gasdermin-B (GSDMB), gasdermin-C (GSDMC), gasdermin-D (GSDMD), and gasdermin-E (GSDME, also known as DFNA5) (Broz et al. 2020). Pyroptosis is often divided into classical and nonclassical pathways. The classical pyroptosis pathway is activated by caspase-1 to cleave GSDMD. Unlike the classical pyroptosis pathway, the nonclassical pyroptosis pathway is activated by caspase-4, caspase-5, and caspase-11 to cleave GSDMD (Kovacs and Miao 2017; Opdenbosch and Lamkanfi 2019). Furthermore, Wang’s study demonstrated that chemotherapy drugs induce pyroptosis through caspase-3 cleavage of GSDME (Wang et al. 2017). When these cleaved gasdermin proteins bind to cardiolipin, phosphatidylinositol, and membrane lipids, the complex is located in the cell membrane and forms 10 to 20 nm pores (Ding et al. 2016; Feng et al. 2018). Cell contents will slowly be released through membrane pores and trigger an amplified inflammatory response. The cells gradually flatten and produce 1–5 μm apoptotic vesicles (scorched vesicles), and the cells gradually expand until the plasma membrane ruptures, with the characteristics of nuclear condensation and chromatin DNA fragmentation (Frank and Vince 2019; Zhang et al. 2018). Studies have reported that the efficient proinflammatory effect of pyroptosis is related to the regulation of the tumor immune microenvironment (Tang et al. 2020; Orning et al. 2019). Defective GSDMD expression is associated with a significant decrease in the number and activity of CD8+ T lymphocytes (Xi et al. 2019). In addition, one study also proved that pyroptosis plays a crucial role in the antitumor function of NK cells (Zhang et al. 2020). Pyroptosis plays a critical role in developing tumors and the antitumor process.

Caspase family proteins and Gasdermin family proteins are closely related to pyroptosis (Opdenbosch and Lamkanfi 2019; Shi et al. 2017). Therefore, analyzing the levels of PRGs and their relationship with the survival of HCC patients is of great value. Moreover, the new prognostic model constructed by PRGs can provide more guidance for targeted therapy. Therefore, our study intends to explore the predictive value of these genes by analyzing the expression level of PRGs between HCC tissues and nontumor tissues and to investigate the correlation between pyroptosis and the tumor immune microenvironment to provide potential therapeutic guidance for HCC targeting and immunotherapy.

## Materials and methods

### Data sources

The RNA sequencing (RNA-seq) data of 374 HCC patients and their clinicopathological parameters were downloaded from the cancer genome atlas (TCGA) public database. (https://portal.gdc.cancer.gov). In addition, we obtained RNA-seq data and clinicopathological features from the Gene Expression Omnibus (GEO) database (https://www.ncbi.nlm.nih.gov/geo/, ID: GSE10186) for validation.

### Identification of differentially expressed PRGs

According to previous research reports, we extracted 35 pyroptosis-related genes (PRGs) (Karki and Kanneganti 2019; Man and Kanneganti 2015; Xia et al. 2019; Ye et al. 2021). The expression of PRGs from the TCGA was analyzed to identify the differentially expressed genes (DEGs) between nontumor and tumor samples. The expression data were normalized to fragment per kilobase million (FPKM) values before comparison. The DEGs with a p value < 0.05 were considered significant and were marked as follows: * when p < 0.05, ** when p < 0.01, and *** when p < 0.001. The DEGs were identified by the “limma” package of R software. The Search Tool for the Retrieval of Interacting Genes (STRING, version 11.0, https://string-db.org/) was used to investigate the protein–protein interaction (PPI) network to determine the interaction of pyroptosis-related genes in this study. The DEGs are shown in Additional file 1: Table S1.

### Development and validation of the PRGs prognostic model

To avoid omissions, 0.2 was set as the cutoff p value, and survival-related genes were recognized for subsequent analysis. Cox regression analysis was used to evaluate the prognostic value of the PRGs in the TCGA dataset. To select the most relevant genes for pyroptosis in the prognosis of HCC patients, we used the least absolute shrinkage and selection operator (LASSO)-Cox regression model to screen the candidate genes and establish a predictive model. LASSO-Cox regression was implemented through the glmnet package of R software. The risk score was calculated according to the centralized and standardized HCC mRNA expression data in the TCGA dataset.

$$\text{Risk}\;\text{score}={\sum_i^k}Xi\times Yi\;(X{:}\;\text{coefficients},\;Y{:}\;\text{gene}\;\text{expression}\;\text{level}).$$  

HCC patients were divided into high-risk and low-risk groups based on the median risk score, and the overall survival (OS) between the two groups was analyzed. To make the model more convincing, this study also utilized the HCC cohort in the GEO database (GSE10186) for validation. The expression of each PRG was also normalized, and the risk score was then calculated by the above formula. HCC patients in the GSE10186 cohort were also grouped into high-risk and low-risk groups according to the median risk score, and the OS between the two groups was compared. Principal component analysis (PCA) based on the PRG signature was performed by the “prcomp” function in the “stats” R package. The ROC curves were plotted by the “time-ROC”, “survminer” and “survival” packages of R.

### Prognostic evaluation of the risk score

The clinicopathological features from the TCGA cohort and the GEO cohort were downloaded and subsequently analyzed. Univariate and multivariate Cox regression models were used to examine independent risk factors for HCC patients. According to the median risk score, the HCC patients in the TCGA database were divided into high-risk and low-risk groups, and the functional enrichment analysis of the DEGs between the two groups was evaluated. The DEGs were screened based on the criteria of log2-fold change ≥ 2 and FDR < 0.05. The “clusterProfiler” package of R was used to investigate Gene Ontology (GO) and Kyoto Encyclopedia of Genes and Genomes (KEGG) analyses. The scores of infiltrating immune cells and the activity of immune-related pathways were analyzed by single-sample gene set enrichment analysis (ssGSEA), which was performed by the “gsva” package.

### Statistics

The Mann–Whitney U test was used to analyze the expression levels of genes between the nontumor tissues and tumor tissues and compare immune cell infiltration and immune pathway activation between groups. The chi-square test was applied to compare the categorical variables. The LASSO regression was used to calculate coefficients of the prognostic signature. The Kaplan–Meier method and a log-rank test were used to compare survival rates between subgroups. The correlation analysis was performed by the Pearson test. Univariate and multivariate Cox regression models were conducted to examine the independent risk factors for the model. All statistical analyses were performed with R software (version 4.1.1) and IBM SPSS (version 26.0, IBM Corporation, Armonk, New York, USA). Two-tailed p < 0.05 was considered statistically significant in all tests.

## Results

### Identification of DEGs between tumor and nontumor samples

**Fig. 1:**
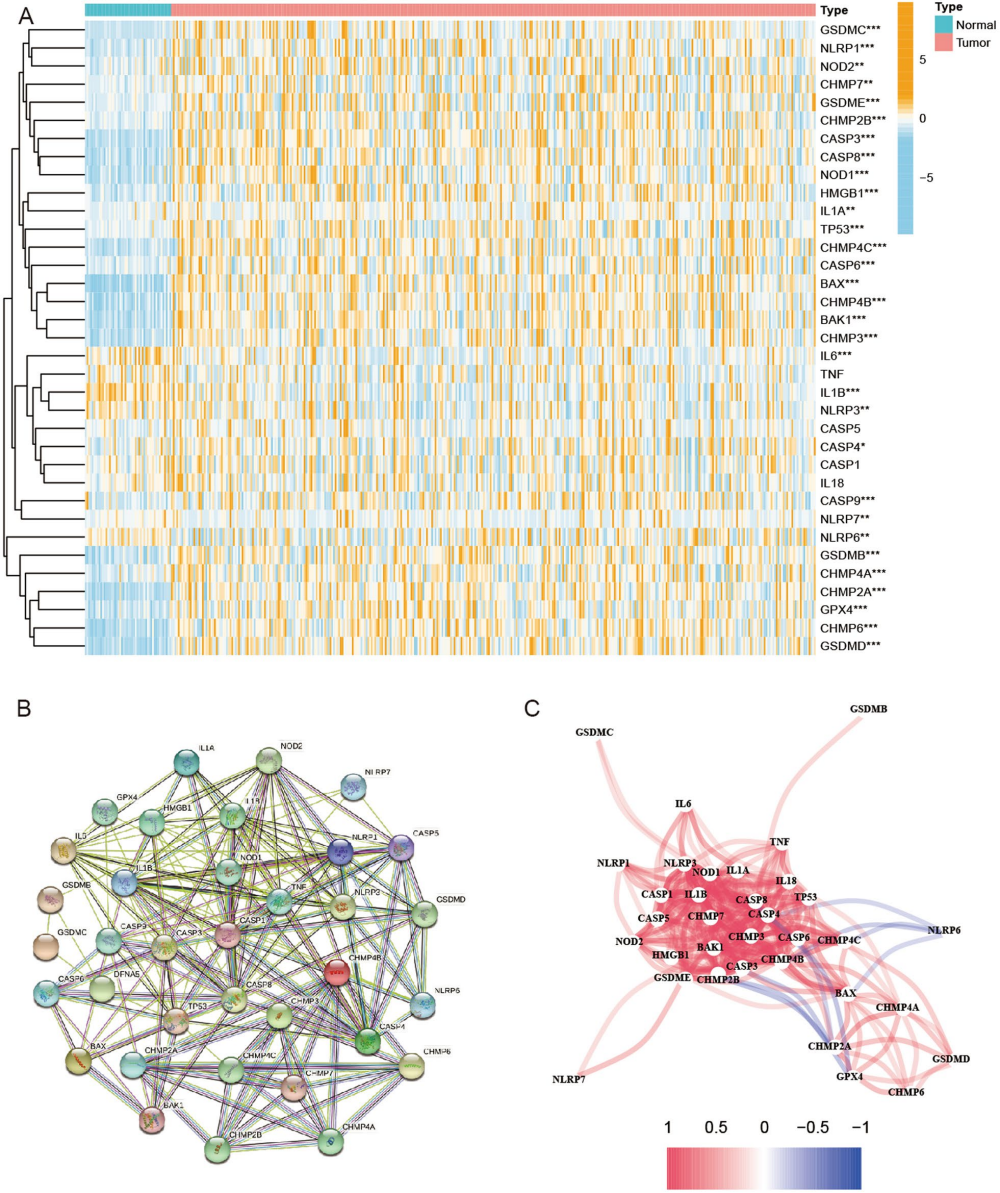
Expression of the 35 PRGs and the interactions among them. **A** Heatmap (blue: low expression level; orange: high expression level) of the PRGs between the nontumor (N, blue) and tumor samples (T, red). P values were shown as: **p < 0.01; ***p < 0.001. **B** PPI network showing the interactions of the PRGs (interaction score = 0.4). **C** The correlation network of the PRGs (red line: positive correlation; blue line: negative correlation. The depth of the colors reflects the strength of the relevance)

The expression levels of 35 PRGs were analyzed between nontumor and tumor samples in the TCGA database. As a result, 31 DEGs were identified (all P < 0.05). Twenty-eight of them (BAK1, BAX, CASP3, CASP4, CASP6, CASP8, CASP9, CHMP2A, CHMP2B, CHMP3, CHMP4A, CHMP4B, CHMP4C, CHMP6, CHMP7, GSDMB, GSDMC, GSDMD, GSDME, HMGB1, IL1A, TP53, GPX4, NLRP1, NLRP6, NLRP7, NOD1, and NOD2) were upregulated, while 3 of these genes (IL6, IL1B, and NLRP3) were downregulated. The expression levels of PRGs are shown in Fig. 1A. In addition, a protein–protein interaction (PPI), which was set at 0.4 (medium confidence) as an interaction score, was analyzed to explore the correlations of the PRGs (Fig. 1B). CASP1, CASP3, CASP4, CASP5, CASP8, IL1B, NLRP3, and CHMP7 were considered hub genes. The correlation network, including all PRGs, is shown in Fig. 1C.

### Classification of HCC patients based on the PRGs

**Fig. 2:**
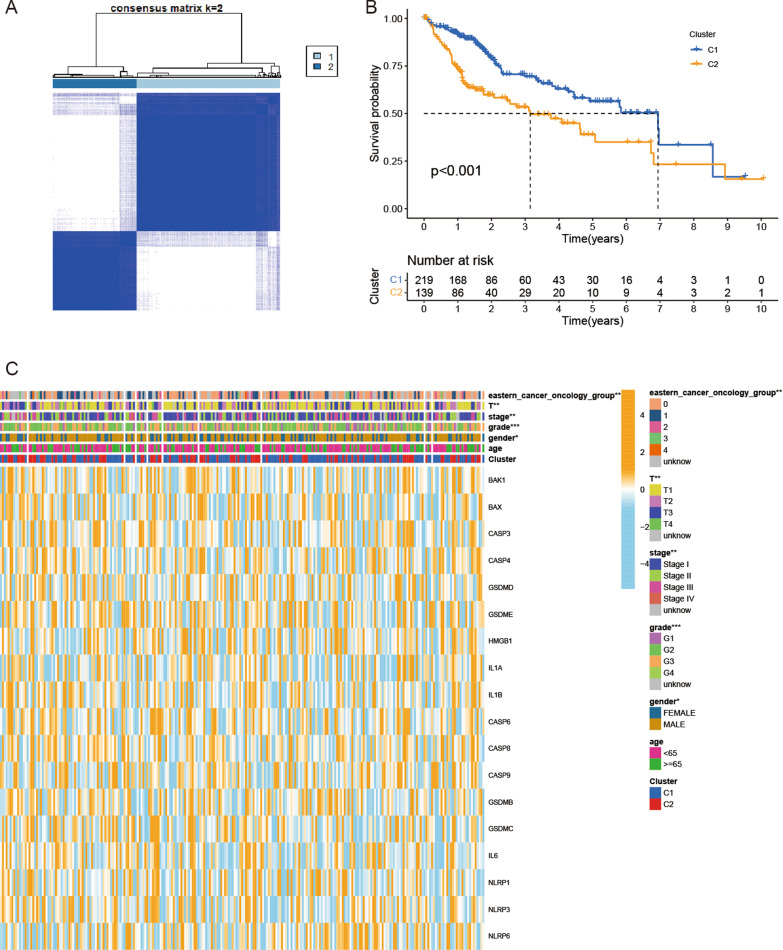
Tumor classification based on the PRGs. **A** 374 HCC patients were grouped into two clusters according to the consensus clustering matrix (k = 2). **B** Kaplan–Meier OS curves for the two clusters. **C** Heatmap and the clinicopathological features of the two clusters classified by these PRGs

To investigate the relationship between the expression of the 35 PRGs and HCC, we performed a consensus clustering analysis of HCC patients in the TCGA database. We found that by increasing the clustering variable (k) from 2 to 10, when k = 2, the intragroup correlations were the highest, which showed that 374 HCC patients could be divided into two clusters according to the 35 PRGs (Fig. 2A). The OS of cluster 1 was better than that of cluster 2 (p < 0.001, Fig. 2B). In addition, the gene expression profile and clinicopathological parameters, including age (< 65 or ≥ 65 years), sex, tumor grade (G1–G4), tumor stage (I–IV), T classification (T1–T4), and Eastern Cancer On-cology Group (ECOG) (0–4), are illustrated in a heatmap. Sex (p < 0.05), tumor grade (p < 0.001), tumor stage (p < 0.01), T classification (p < 0.01), and ECOG (p < 0.01) were significantly different between the two clusters (Fig. 2C).

### Establishment of a prognostic gene model based on the TCGA cohort

**Fig. 3:**
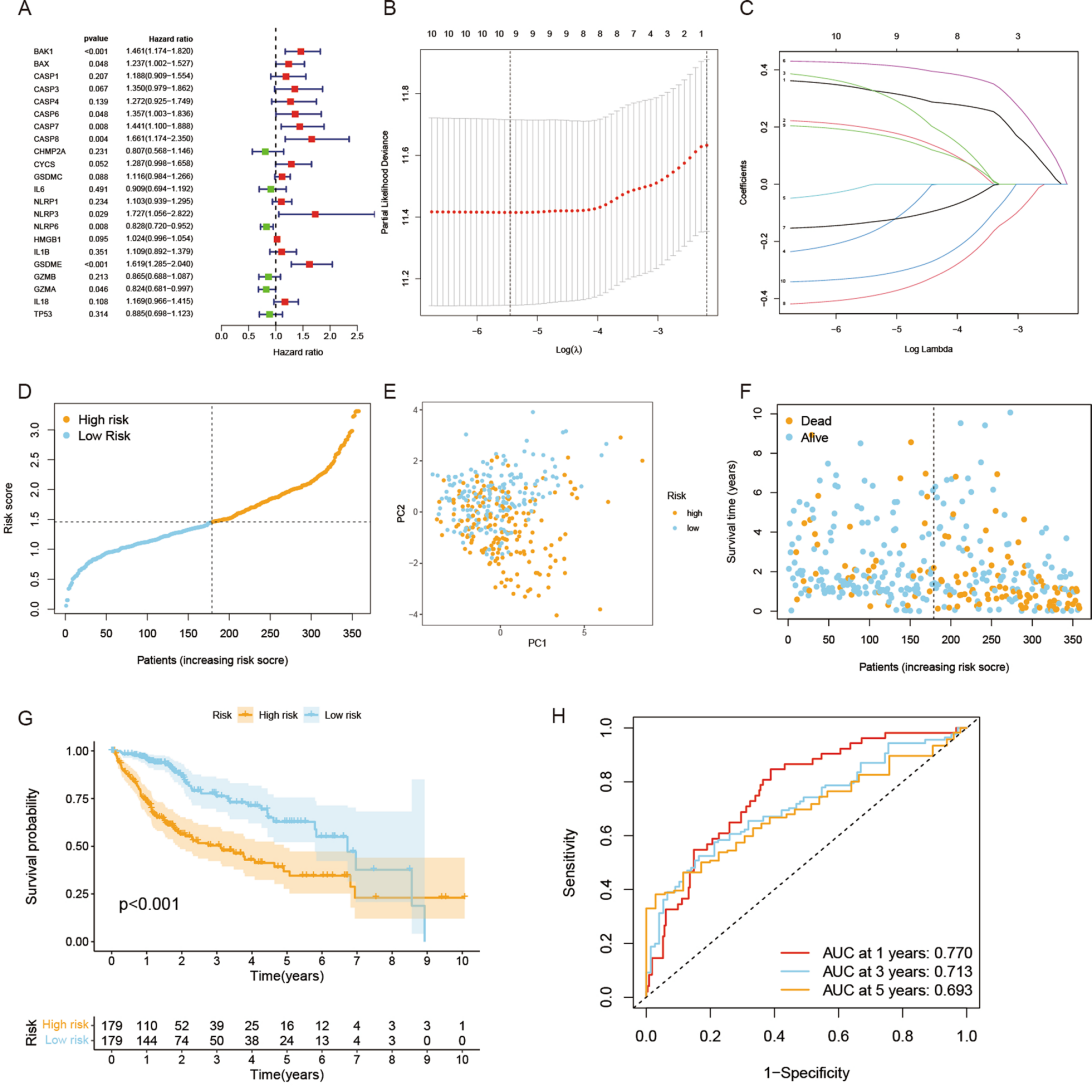
Construction of the risk signature in the TCGA cohort. **A** Univariate Cox regression analysis of HCC for each PRG and 10 genes with P < 0.2. **B** Cross-validation for tuning the parameter selection in the LASSO regression. **C** LASSO regression of the 10 OS-related genes. **D** Distribution of patients based on the risk score. **E** PCA plot for HCCs based on the risk score. **F** The survival status for each patient (low-risk population: on the left side of the dotted line; high-risk population: on the right side of the dotted line). **G** Kaplan–Meier curves for the OS of patients in the high- and low-risk groups. **H** ROC curves demonstrated the predictive efficiency of the risk score

A total of 374 HCC patients with complete survival data from the TCGA database were selected for analysis. Survival-related genes were screened by univariate Cox regression analysis. A total of 22 genes that met the p < 0.5 criteria, including BAK1, BAX, CASP1, CASP3, CASP4, CASP6, CASP7, CASP8, CHMP2A, CYCS, GSDMC, GSDME, IL1B, IL6, IL18, NLRP1, NLRP3, NLRP6, HMGB1, GZMA, GZMB, and TP53, were reserved for subsequent analysis. Of these, 16 genes (BAK1, BAX, CASP1, CASP3, CASP4, CASP6, CASP7, CASP8, CYCS, GSDMC, GSDME, IL1B, IL18, NLRP1, NLRP3, and HMGB1) were associated with an increased risk of HRs > 1, while the other 6 genes (CHMP2A, IL6, NLRP6, GZMA, GZMB, and TP53) were associated with HRs < 1 (Fig. 3A). We generated a 10-gene signature utilizing least absolute shrinkage and selection operator (LASSO) Cox regression (Fig. 3B, C). The formula for calculating the risk score is as follows: Risk score = (0.329 * BAK1exp.) + (0.196 * BAXexp.) + (0.332 * CASP1exp.) + (− 0.171 * CASP4exp.) + (− 0.003 * CASP6exp.) + (0.42 * GSDMEexp.) + (− 0.392 * GZMAexp.) + (− 0.133 * GZMBexp.) + 179 * IL18exp.) + (− 0.322 * TP53exp.).

HCC patients were divided into a low-risk group and a high-risk group according to the median risk score (Fig. 3D). The results showed that HCC patients in different groups were well separated into two clusters through principal component analysis (PCA) (Fig. 3E). In addition, high-risk HCC patients had poorer survival than low-risk patients (p < 0.001) (Fig. 3F, G). The predictive model constructed by the risk score was evaluated by time-dependent receiver operating characteristic (ROC) analysis. The areas under the ROC curve (AUCs) at 1 year, 3 years, and 5 years were 0.770, 0.713, and 0.693, respectively (Fig. 3H).

### Validation of the risk signature

**Fig. 4:**
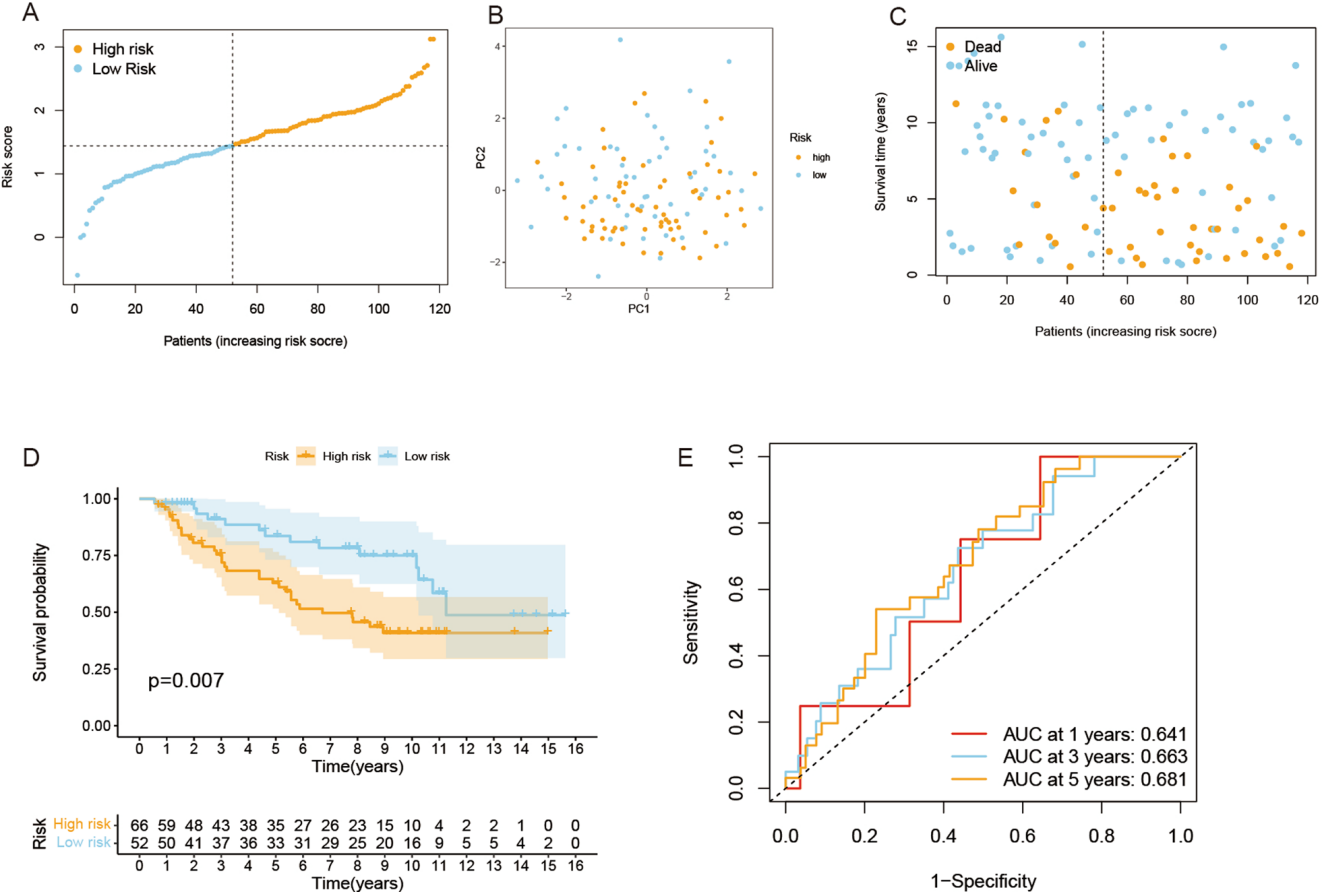
Validation of the risk model in the GEO cohort. **A** Distribution of patients in the GEO cohort based on the median risk score in the TCGA cohort. **B** PCA plot for HCCs. **C** The survival status for each patient (low-risk population: on the left side of the dotted line; high-risk population: on the right side of the dotted line). **D** Kaplan–Meier curves for comparison of the OS between low- and high-risk groups. **E** Time-dependent ROC curves for HCCs

A total of 118 HCC patients from the GEO database (GSE10186) were selected as the validation set. Gene expression levels were normalized by the “Scale” function for subsequent analysis. A total of 118 HCC patients in the GEO cohort were divided into high-risk and low-risk groups according to the median risk score obtained from the TCGA cohort (Fig. 4A). The PCA displayed a moderate result between the two groups (Fig. 4B). Similarly, HCC patients in the high-risk group had poorer survival (p = 0.007) and higher death rates than those in the low-risk group (Fig. 4C, D). This prognostic model also had a moderate predictive capability in the GEO database. The ROC curve of the validation group revealed the results of 1-year, 3-year, and 5-year OS with AUCs of 0.641, 0.663, and 0.681, respectively (Fig. 4E).

### Evaluation of independent prognostic value of the risk model

**Fig. 5:**
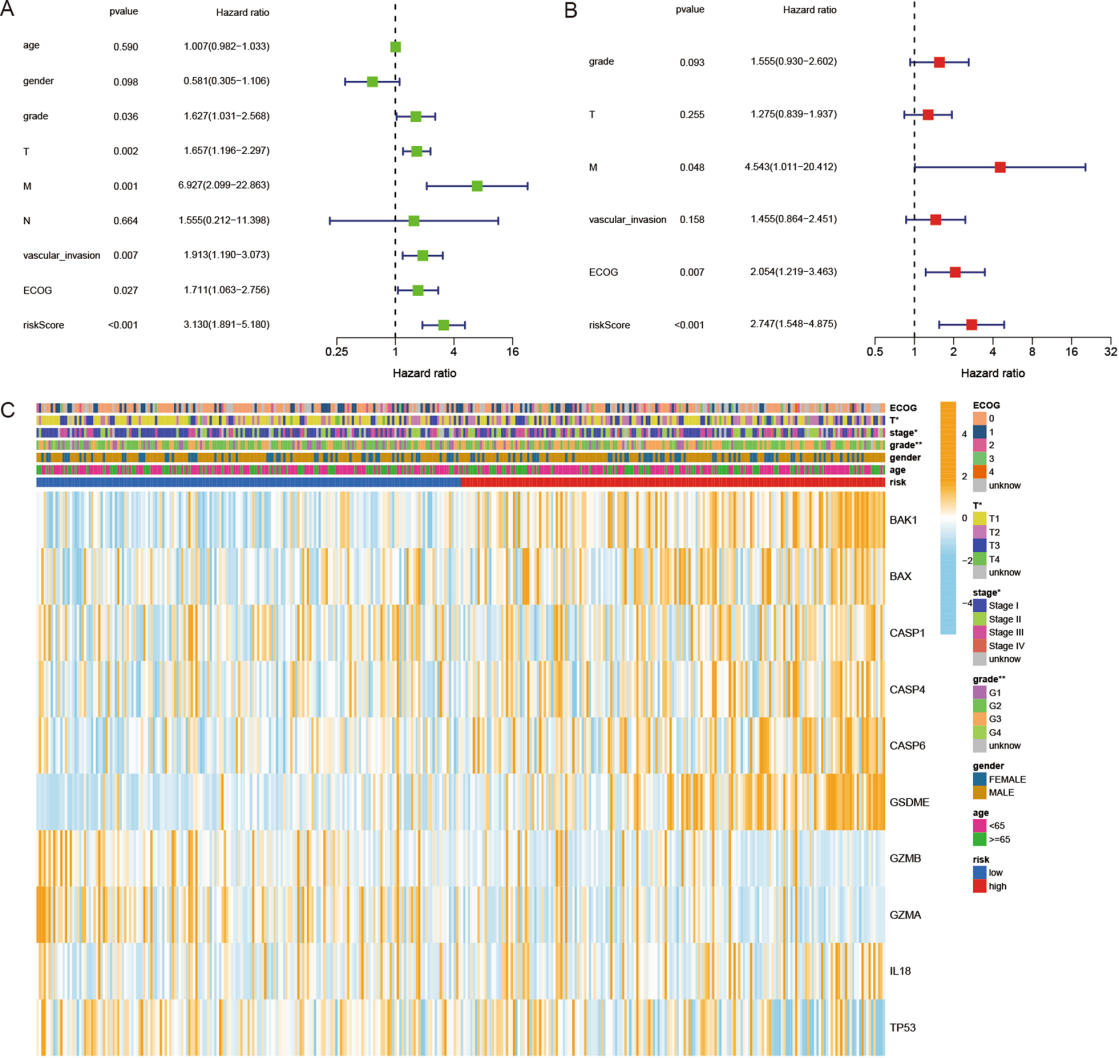
Univariate and multivariate Cox regression analyses for the risk score. **A** Univariate analysis for the TCGA cohort. **B** Multivariate analysis for the TCGA cohort. **C** Heatmap (blue: low expression; orange: high expression) for the connections between clinicopathologic characteristics and the risk groups (*p < 0.05, **p < 0.01)

We performed univariate and multivariable Cox regression analyses to investigate the independent prognostic factors for HCC patients. The results showed that tumor grade (HR = 1.627, 95% CI 1.031–2.568), T classification (HR = 1.657, 95% CI 1.196–2.297), M classification (HR = 6.927, 95% CI 2.099–22.863), vascular invasion (HR = 1.913, 95% CI 1.19–3.073), ECOG (HR = 1.711, 95% CI 1.063–2.756), and risk score (HR = 3.13, 95% CI 1.891–5.18) were prognostic factors in the TCGA cohort by univariate Cox regression analysis (Fig. 5A). By multivariate analysis, M classification (HR = 4.543, 95% CI 1.011–20.412), ECOG (HR = 2.054, 95% CI 1.219–3.463), and risk score (HR = 2.747, 95% CI 1.548–4.875) were independent factors for HCC patients (Fig. 5B). Moreover, a clinicopathological information heatmap was developed based on the TCGA cohort, which showed that HCC patients in the high-risk and low-risk groups showed a significant correlation with T classification (p < 0.05), tumor stage (p < 0.05), and tumor grade (p < 0.01) (Fig. 5C).

### Functional analyses based on the risk model

**Fig. 6:**
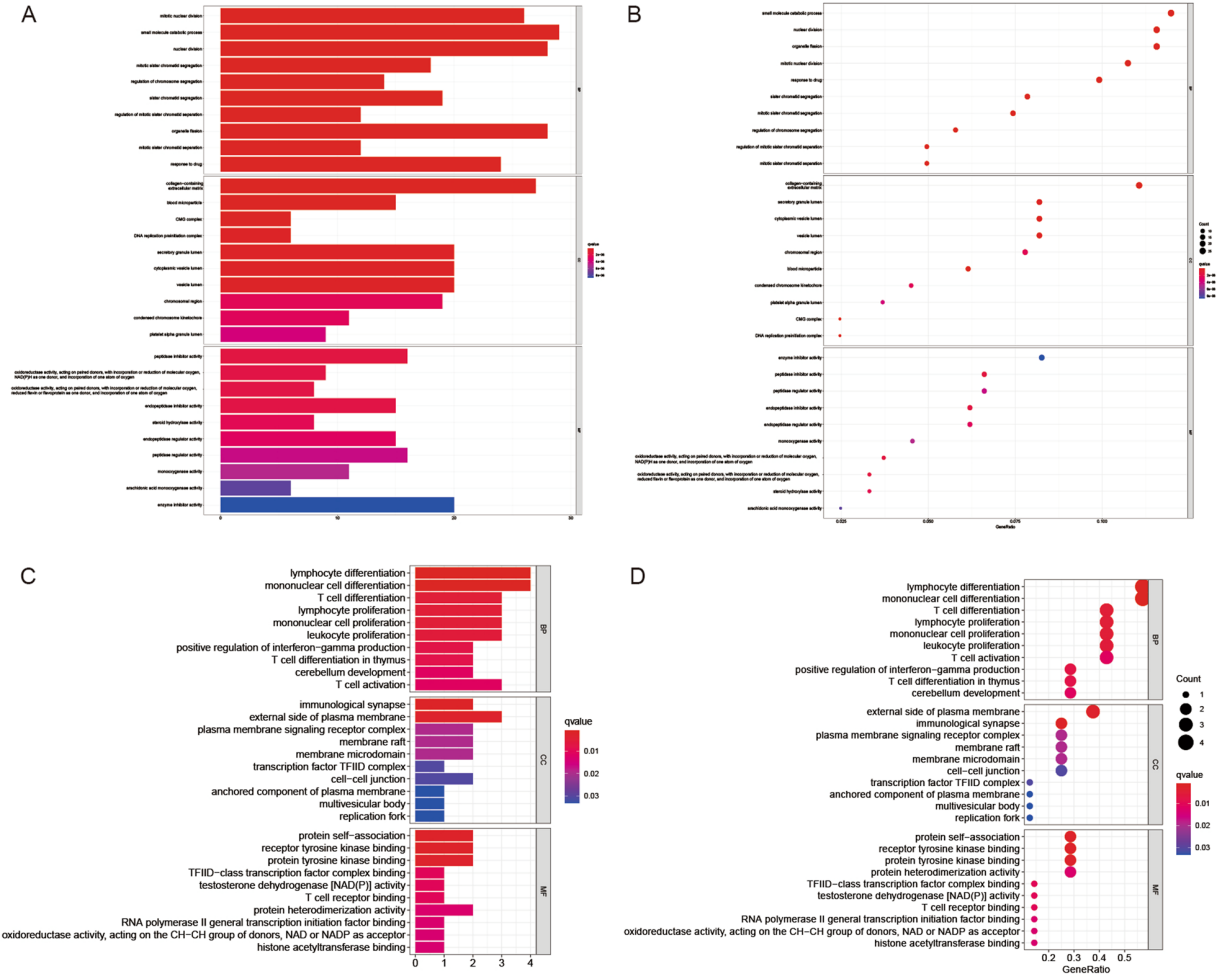
Functional analysis based on the PRGs between the two-risk groups in the TCGA and GEO cohort. **A** Bubble graph for GO enrichment in the TCGA cohort (the larger bubble means the more genes enriched, and the increasing depth of red means the differences were more obvious; q-value: the adjusted p value). **B** Barplot graph for KEGG pathways in the TCGA cohort (the longer bar means the more genes enriched, and the increasing depth of red means the differences were more obvious). **C** Bubble graph for GO enrichment in the GEO cohort. **D** Barplot graph for KEGG pathways in the GEO cohort

To explore the differences in gene functions and gene enrichment between high-risk and low-risk groups according to the risk model, we identified a total of 244 DEGs, which were screened with the criteria of FDR < 0.05 and |log2FC | > 0.585. This analysis was completed by the “limma” package of R. The investigation revealed that the upregulated and downregulated genes in the high-risk group were 144 and 100, respectively (Additional file 2: Table S2). Then, these DEGs were subjected to gene ontology (GO) enrichment analysis and Kyoto Encyclopedia of Genes and Genomes (KEGG) pathway analysis, which indicated that the DEGs were mainly related to inflammatory cell chemotaxis, chemokine-mediated signaling pathways, and immune responses (Fig. 6A–D).

### Comparison of immune activity among different risk groups

**Fig. 7:**
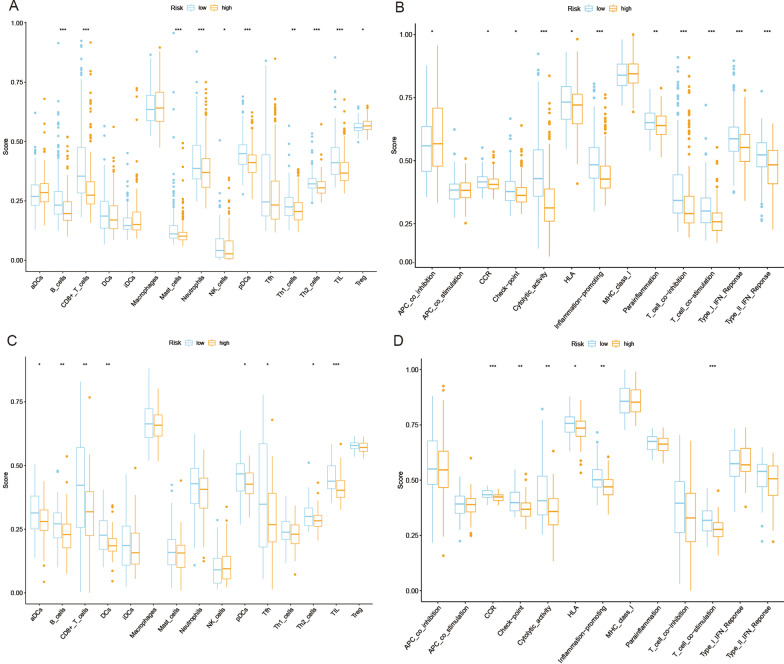
Comparison of the ssGSEA scores for immune cells and immune pathways. **A**, **B** Comparison of the enrichment scores of 15 types of immune cells and 13 immune-related pathways between the low- (blue box) and high-risk (orange box) groups in the TCGA cohort. **C**, **D** Comparison of tumor immunity between the low- (blue box) and high-risk (orange box) groups in the GEO cohort (*p < 0.05; **p < 0.01; ***p < 0.001)

We performed ssGSEA for further functional analysis and compared the enrichment scores of 15 immune cells and the activity of 13 immune-related pathways in the TCGA and GEO databases. In the TCGA cohort (Fig. 7A), compared with the low-risk group, the high-risk group had lower levels of immune cell infiltration, especially B cells, CD8+ T cells, mast cells, neutrophils, natural killer (NK) cells, plasmacytoid dendritic cells (pDCs), helper T (Th) cells (Th1 and Th2 cells), tumor-infiltrating lymphocytes (TILs) and regulatory T (Treg) cells. In the TCGA cohort, the antigen-presenting cell (APC) coinhibition pathway was less active in the low-risk group than in the high-risk group, while the other 10 pathways, including chemokine receptor (CCR), checkpoint, cytolytic activity, human leukocyte antigen (HLA), inflammation-promoting, parainflammation, T cell coinhibition, T cell costimulation, type I interferon (IFN) response, and type II IFN response, were less active in the high-risk group than in the low-risk group (Fig. 7B). In the GEO cohort, the levels of immune cell infiltration of aDCs, B cells, CD8+ T cells, dendritic cells (DCs), pDCs, follicular helper T cells (Tfhs), Th2 cells, and TILs were significantly lower in the high-risk group than in the low-risk group. In addition, immune-related pathways, including CCR, checkpoint, cytolytic activity, HLA, inflammation-promoting, and T cell costimulation, showed less active levels in the high-risk group than in the low-risk group (Fig. 7C, D).

### Establishment of a prognostic nomogram for HCC patients

**Fig. 8:**
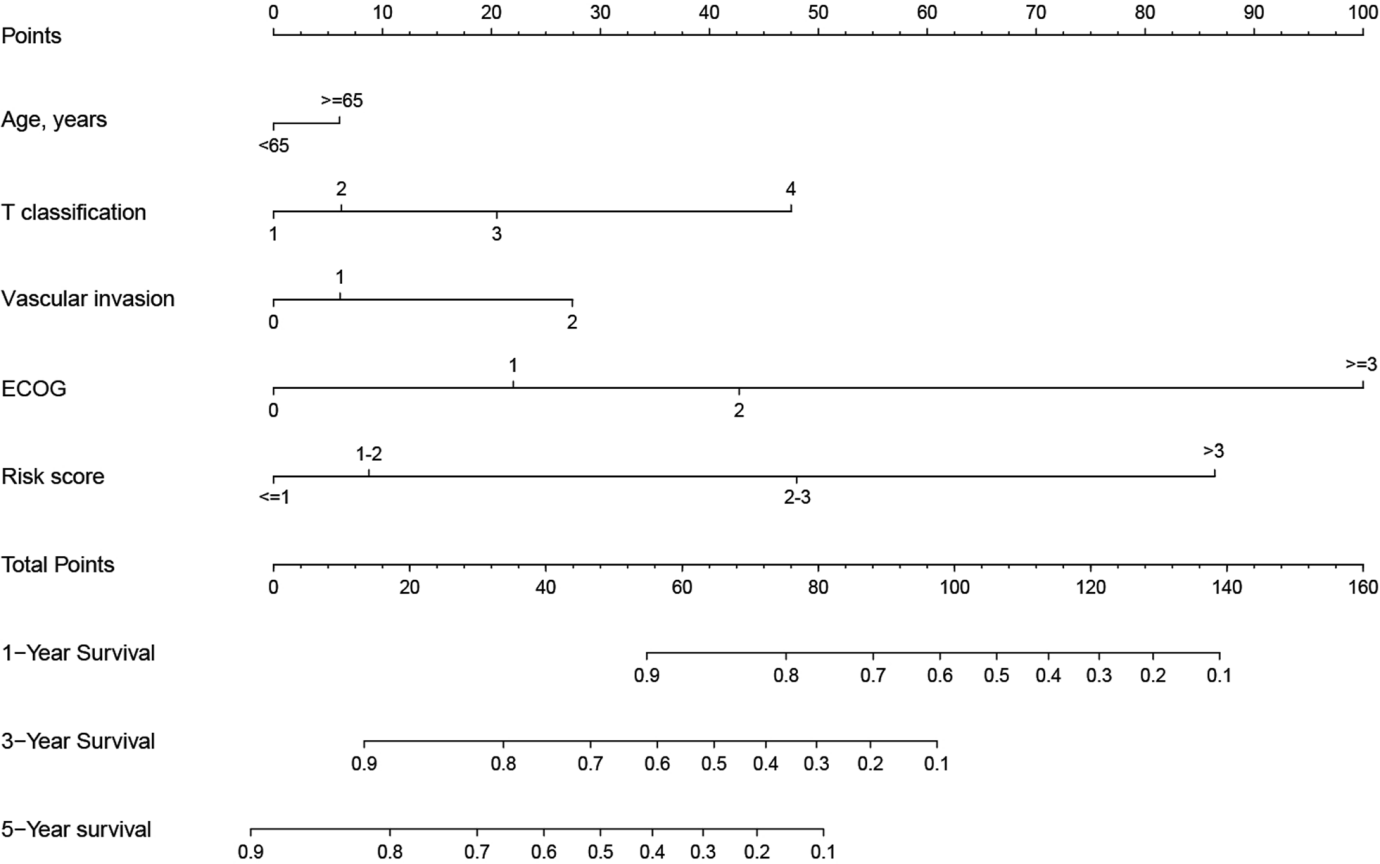
Prognostic nomogram was established by combining clinicopathological parameters and risk score

We generated a new prognostic nomogram based on age, T classification, vascular invasion, ECOG, and risk score to predict HCC patient survival (Fig. 8). The results showed that the nomogram could systematically predict patient OS at 1, 3, and 5 years.

Patients with a higher score had a lower probability of survival. For instance, a case of a 65-year-old male patient with T3, vascular invasion grade 0, ECOG 1, and risk score equal to 2 would score a total of 65 points (5 points for age, 20 points for T classification, 0 points for vascular invasion, 20 points for ECOG, and 10 points for risk score). For this case, the predicted probability of 1-year, 3-year, and 5-year survival was 85.0%, 50%, and 28.0%, respectively.

## Discussion

Our study examined the expression levels of genes reported in the literature to be associated with pyroptosis in HCC and nontumor samples and found that most of these PRGs were differentially expressed. In addition, the two clusters generated by consensus cluster analysis according to the DEGs had significant differences in common clinicopathological features, such as sex, T classification, tumor stage, tumor grade, and ECOG. To further evaluate whether these PRGs have prognostic value in HCC patients, we developed a risk signature composed of 10 genes by Cox univariate and LASSO Cox regression analysis and validated its good performance in the GEO database as external data. Moreover, functional analysis revealed that the DEGs between different risk groups were associated with immune-related pathways. Additionally, analysis of immune cell infiltration and activation pathways showed that the level of infiltrating immune cells and the activity of immune-related pathways in the high-risk group were lower than those in the low-risk group.

Pyroptosis is one type of programmed cell death (Xue et al. 2019). Studies have found that pyroptosis not only plays a vital role in systemic inflammatory response syndrome but also participates in the development and treatment of tumors (Chen et al. 2018; Erkes et al. 2020). First, tumor cells can release a large number of inflammatory factors and immune-related antigens after pyroptosis under various types of stimulation, which may become a new potential therapeutic target (Xia et al. 2019). In addition, normal cells are stimulated by abundant inflammatory factors released by pyroptosis and may transform into malignant cells (Karki and Kanneganti 2019). In breast cancer, the occurrence of pyroptosis has been proven to be associated with tumor chemotherapy drugs (Wang et al. 2017). However, the relationship between PRGs, the development of HCC, and the survival of patients is still unclear. In this study, a signature consisting of 10 PRGs (BAK1, BAX, CASP1, CASP4, CASP6, GSDME, GZMA, GZMB, IL18, and TP53) was selected based on the PRGs reported in the literature (Karki and Kanneganti 2019; Man and Kanneganti 2015; Xia et al. 2019; Ye et al. 2021), which has the capability to predict the survival of HCC patients.

BAK1 and BAX are closely related to cell death, especially apoptosis, and they are regarded as important regulators of apoptosis (Flores-Romero et al. 2020; Westphal et al. 2014). Caspase family genes are associated with cell death pathways and participate in the regulation of cell growth, differentiation, and apoptosis (Opdenbosch and Lamkanfi 2019; Shi 2002). Caspase 1, Caspase 4, and Caspase 6 belong to the caspase family and were confirmed to be related to pyroptosis in our study. Caspase 1 participates in the pyroptosis signaling pathway and induces pyroptosis by cleaving gasdermin proteins (Shi et al. 2017). Moreover, these genes can cleave and activate interleukin-1 (IL1), which is a cytokine involved in inflammation, septic shock, and wound healing (Schneider et al. 2017). Previous studies have suggested that GSDME (also known as DFNA5) is related to deafness (Busch-Nentwich et al. 2004; Camp et al. 1995). Recent studies have demonstrated that GSDME is involved in chemotherapy-induced pyroptosis (Wang et al. 2017; Hu et al. 2020; Jiang et al. 2020). Caspase 3 can cleave GSDME after activation, and cleaved GSDME can form a complex and bind to the cell membrane to cause pyroptosis (Wang et al. 2017). It is noteworthy that some studies have suggested that apoptosis and pyroptosis are not entirely opposite processes. Under certain conditions, apoptosis can be transformed into pyroptosis (Feng et al. 2018; Jiang et al. 2020; Aglietti and Dueber 2017). Wang’s research indicates that cancer cells treated with chemotherapeutic drugs can undergo apoptosis and pyroptosis, due to the primary expression of GSDME (Wang et al. 2017). Chemotherapy drugs are more likely to trigger pyroptosis in cells with high GSDME expression. Granzyme A (GZMA) is related to apoptosis, autophagy, and pyroptosis pathways. When GZMA is delivered to target cells, it can act by catalyzing the lysis of GSDMB, thereby triggering pyroptosis and target cell death (Martinvalet et al. 2008; Zhou et al. 2020). Similarly, when granzyme B (GZMB) is delivered into the target cells, it can act by catalyzing the lysis of GSDME, releasing the pore-forming moiety of GSDME and triggering pyroptosis and target cell death (Zhang et al. 2020). IL18 is a proinflammatory cytokine that is mainly involved in the immune response of polarized T helper cell 1 (Th1) cells and natural killer (NK) cells. Inactive IL18 precursors are processed into their active form by caspase-1 and can stimulate interferon γ and regulate helper T cell (Th) 1 and Th2 responses (Kaplanski 2018). TP53, a coding gene that can encode tumor suppressor proteins, can induce apoptosis, cell cycle arrest, DNA repair, etc. (Bieging et al. 2014; Sharma et al. 2017).

Although current studies have found some similarities and cross-effects between pyroptosis and apoptosis (Frank and Vince 2019; Tang et al. 2020), research on pyroptosis still needs to be further explored. A variety of cell death patterns may coexist and interact during tumor development (Fritsch et al. 2019). For instance, 7 genes in our model (BAK1, BAX, CASP1, CASP4, CASP6, GZMA, and GZMB) are critical regulators of apoptotic pathways. We investigated the DEGs between different risk groups and discovered that DEGs are mainly involved in the immune response and inflammatory cell chemotaxis, indicating that dead cells can induce a robust inflammatory response. According to the results of the GO and KEGG analyses, pyroptosis may regulate the tumor immune microenvironment.

Studies have shown that pyroptosis is related to immunity (Tang et al. 2020; Zhang et al. 2020). After tumor cells undergo pyroptosis, the expression of immunogenicity increases, which can improve the efficacy of immunotherapy (Hou et al. 2020). Based on data from the TCGA and GEO databases, our research demonstrates that the infiltration levels of some critical immune cells, such as B cells, CD8+ T cells, and TILs, were significantly lower in the high-risk group than in the low-risk group. Moreover, the levels of immune-related pathways, including cytolytic activity, HLA, and T cell costimulation, were less active in the high-risk group than in the low-risk group. Studies by Ye et al. (2021), Ju et al. (2021), Shao et al. (2021), Isik et al. (2016, 2021). The risk model constructed by the signature composed of PRGs can predict the prognosis of cancer patients, and PRGs are related to tumor immunity.

Currently, there are few studies on the mechanism of pyroptosis in HCC. GSDME, one of the members of the gasdermin family, may be the executor of pyroptosis, and 10 genes related to cell death regulation were identified in our research. We examined the prognostic value of the PRGs, which provided theoretical support for in-depth analysis. However, due to the lack of basic experimental validation, how these related genes function in HCC is unclear and is worthy of further exploration.

## Conclusions

In conclusion, our research shows that most PRGs are differentially expressed between HCC and nontumor samples, and pyroptosis is closely related to HCC. In addition, the risk scores calculated in this study according to the 9 PRGs can be considered independent risk factors for predicting HCC in the TCGA and GEO databases. The DEGs between different risk groups are associated with tumor immunity. Therefore, this study can be used to identify novel predictive markers for the prognosis of HCC patients and provides an important basis for future research on the relationship between PRGs and HCC immunity.

## Supplementary Information

**Additional file 1: Table S1.** The expression levels of pyroptosis-related genes.

**Additional file 2: Table S2.** Differentially expressed genes.

## Data Availability

Publicly available datasets were analyzed in this study, these can be found in The Cancer Genome Atlas (https://portal.gdc.cancer.gov/) and the Gene Expression Omnibus (https://www.ncbi.nlm.nih.gov/geo/). Additional data associated with this article, named “Table S1” and “Table S2” have been uploaded.

## Abbreviations

APC: Antigen-presenting cell
AUC: Areas under the ROC curve
CCR: Chemokine receptor
DCs: Dendritic cells
DEGs: Differentially expressed genes
ECOG: Eastern Cancer On-cology Group
FPKM: Fragment per kilobase million
GEO: Gene Expression Omnibus
GO: Gene ontology
GSDMA: Gasdermin-A
GSDMB: Gasdermin-B
GSDMC: Gasdermin-C
GSDMD: Gasdermin-D
GSDME: Gasdermin-E
GZMA: Granzyme A
GZMB: Granzyme B
HCC: Hepatocellular carcinoma
HLA: Human leukocyte antigen
ICI: Immune checkpoint inhibitor
KEGG: Kyoto Encyclopedia of Genes and Genomes
LASSO: The least absolute shrinkage and selection operator
NK cell: Natural killer cells
OS: Overall survival
PCA: Principal component analysis (PCA)
PD-1: Programmed cell death protein 1
PD-L1: Programmed cell death receptor ligand 1
pDCs: Plasmacytoid dendritic cells
PPI: Protein–protein interaction
PRGs: Pyroptosis-related genes
ROC: Receiver operating characteristic
ssGSEA: Single-sample gene set enrichment analysis
STRING: Search Tool for the Retrieval of Interacting Genes
TCGA: The cancer genome atlas
Tfhs: Follicular helper T cells
Th cell: Helper T cell
TILs: Tumor-infiltrating lymphocytes
TNF: Type I interferon
Treg: Regulatory T cells

## References

[CR1] Aglietti RA, Dueber EC (2017). Recent insights into the molecular mechanisms underlying pyroptosis and gasdermin family functions. Trends Immunol.

[CR2] Anwanwan D, Singh SK, Singh S, Saikam V, Singh R (2020). Challenges in liver cancer and possible treatment approaches. Biochim Biophys Acta Rev Cancer.

[CR3] Bieging KT, Mello SS, Attardi LD (2014). Unravelling mechanisms of p53-mediated tumour suppression. Nat Rev Cancer.

[CR4] Broz P, Pelegrin P, Shao F (2020). The gasdermins, a protein family executing cell death and inflammation. Nat Rev Immunol.

[CR5] Busch-Nentwich E, Sollner C, Roehl H, Nicolson T (2004). The deafness gene dfna5 is crucial for ugdh expression and HA production in the developing ear in zebrafish. Development.

[CR6] Chen N (2018). Cathepsin B regulates non-canonical NLRP3 inflammasome pathway by modulating activation of caspase-11 in Kupffer cells. Cell Prolif.

[CR7] Chen S, Cao Q, Wen W, Wang H (2019). Targeted therapy for hepatocellular carcinoma: challenges and opportunities. Cancer Lett.

[CR8] Cheng AL, Hsu C, Chan SL, Choo SP, Kudo M (2020). Challenges of combination therapy with immune checkpoint inhibitors for hepatocellular carcinoma. J Hepatol.

[CR9] Ding J (2016). Pore-forming activity and structural autoinhibition of the gasdermin family. Nature.

[CR10] Erkes DA (2020). Mutant BRAF and MEK inhibitors regulate the tumor immune microenvironment via pyroptosis. Cancer Discov.

[CR11] Feng S, Fox D, Man SM (2018). Mechanisms of gasdermin family members in inflammasome signaling and cell death. J Mol Biol.

[CR12] Flores-Romero H, Ros U, Garcia-Saez AJ (2020). Pore formation in regulated cell death. EMBO J.

[CR13] Forner A, Reig M, Bruix J (2018). Hepatocellular carcinoma. Lancet.

[CR14] Frank D, Vince JE (2019). Pyroptosis versus necroptosis: similarities, differences, and crosstalk. Cell Death Differ.

[CR15] Fritsch M (2019). Caspase-8 is the molecular switch for apoptosis, necroptosis and pyroptosis. Nature.

[CR16] Greten TF, Lai CW, Li G, Staveley-O’Carroll KF (2019). Targeted and immune-based therapies for hepatocellular carcinoma. Gastroenterology.

[CR17] Hou J (2020). PD-L1-mediated gasdermin C expression switches apoptosis to pyroptosis in cancer cells and facilitates tumour necrosis. Nat Cell Biol.

[CR18] Hu L (2020). Chemotherapy-induced pyroptosis is mediated by BAK/BAX-caspase-3-GSDME pathway and inhibited by 2-bromopalmitate. Cell Death Dis.

[CR19] Isik A, Firat D, Soyturk M, Eken H, Ylmaz I (2016). Gallbladder duplication. Gazi Med J.

[CR20] Isik A, Soran A, Grasi A, Barry N, Sezgin E (2021). Lymphedema after sentinel lymph node biopsy: who is at Risk?. Lymphat Res Biol.

[CR21] Jiang M, Qi L, Li L, Li Y (2020). The caspase-3/GSDME signal pathway as a switch between apoptosis and pyroptosis in cancer. Cell Death Discov.

[CR22] Ju A, Tang J, Chen S, Fu Y, Luo Y (2021). Pyroptosis-related gene signatures can robustly diagnose skin cutaneous melanoma and predict the prognosis. Front Oncol.

[CR23] Kaplanski G (2018). Interleukin-18: biological properties and role in disease pathogenesis. Immunol Rev.

[CR24] Karki R, Kanneganti TD (2019). Diverging inflammasome signals in tumorigenesis and potential targeting. Nat Rev Cancer.

[CR25] Kovacs SB, Miao EA (2017). Gasdermins: effectors of pyroptosis. Trends Cell Biol.

[CR26] Llovet JM, Montal R, Sia D, Finn RS (2018). Molecular therapies and precision medicine for hepatocellular carcinoma. Nat Rev Clin Oncol.

[CR27] Man SM, Kanneganti TD (2015). Regulation of inflammasome activation. Immunol Rev.

[CR28] Martinvalet D, Dykxhoorn DM, Ferrini R, Lieberman J (2008). Granzyme A cleaves a mitochondrial complex I protein to initiate caspase-independent cell death. Cell.

[CR29] Orning P, Lien E, Fitzgerald KA (2019). Gasdermins and their role in immunity and inflammation. J Exp Med.

[CR30] Schneider KS, (2017). The inflammasome drives GSDMD-independent secondary pyroptosis and IL-1 release in the absence of Caspase-1 protease activity. Cell Rep.

[CR31] Shao W (2021). The pyroptosis-related signature predicts prognosis and indicates immune microenvironment infiltration in gastric cancer. Front Cell Dev Biol.

[CR32] Sharma G (2017). p53 dependent apoptosis and cell cycle delay induced by heteroleptic complexes in human cervical cancer cells. Biomed Pharmacother.

[CR33] Shi Y (2002). Mechanisms of caspase activation and inhibition during apoptosis. Mol Cell.

[CR34] Shi J, Gao W, Shao F (2017). Pyroptosis: gasdermin-mediated programmed necrotic cell death. Trends Biochem Sci.

[CR35] Tang R (2020). Ferroptosis, necroptosis, and pyroptosis in anticancer immunity. J Hematol Oncol.

[CR36] van Camp G (1995). Localization of a gene for non-syndromic hearing loss (DFNA5) to chromosome 7p15. Hum Mol Genet.

[CR37] Van Opdenbosch N, Lamkanfi M (2019). Caspases in cell death, inflammation, and disease. Immunity.

[CR38] Vibert E, Schwartz M, Olthoff KM (2020). Advances in resection and transplantation for hepatocellular carcinoma. J Hepatol.

[CR39] Villanueva A (2019). Hepatocellular carcinoma. N Engl J Med.

[CR40] Wang Y (2017). Chemotherapy drugs induce pyroptosis through caspase-3 cleavage of a gasdermin. Nature.

[CR41] Westphal D, Kluck RM, Dewson G (2014). Building blocks of the apoptotic pore: how Bax and Bak are activated and oligomerize during apoptosis. Cell Death Differ.

[CR42] Xi G (2019). GSDMD is required for effector CD8(+) T cell responses to lung cancer cells. Int Immunopharmacol.

[CR43] Xia X (2019). The role of pyroptosis in cancer: pro-cancer or pro-“host”?. Cell Death Dis.

[CR44] Xue Y, Enosi Tuipulotu D, Tan WH, Kay C, Man SM (2019). Emerging activators and regulators of inflammasomes and pyroptosis. Trends Immunol.

[CR45] Ye Y, Dai Q, Qi H (2021). A novel defined pyroptosis-related gene signature for predicting the prognosis of ovarian cancer. Cell Death Discov.

[CR46] Zhang Y, Chen X, Gueydan C, Han J (2018). Plasma membrane changes during programmed cell deaths. Cell Res.

[CR47] Zhang Z (2020). Gasdermin E suppresses tumour growth by activating anti-tumour immunity. Nature.

[CR48] Zhou Z (2020). Granzyme A from cytotoxic lymphocytes cleaves GSDMB to trigger pyroptosis in target cells. Science.

