# Supplementary material for: The pyroptosis-related gene signature predicts prognosis and indicates immune activity in hepatocellular carcinoma
Source: Mol Med. 2022 Feb 5;28:16. doi: 10.1186/s10020-022-00445-0 (PMC8818170; doi:10.1186/s10020-022-00445-0)
Supplement: Supplementary file 1 — Additional file 1: Table S1. The expression levels of pyroptosis-related genes. [file 10020_2022_445_MOESM1_ESM.pdf]

Table S1 The expression levels of pyroptosis-related genes

gene	conMean	treatMean	logFC	pValue
BAK1	2.432961	7.457171	1.615915	1.55E-17
BAX	7.321618	21.10099	1.527077	7.52E-23
CASP1	2.356665	2.667478	0.17873	0.697343
CASP3	4.186012	8.440812	1.011805	1.98E-17
CASP4	4.200851	5.106819	0.281743	0.032861
CASP5	0.044708	0.087947	0.976092	0.161364
CHMP2A	33.2192	65.21389	0.973162	9.02E-22
CHMP2B	7.371213	9.876101	0.42204	1.91E-05
CHMP3	2.053975	4.198039	1.031297	4.04E-17
CHMP4A	0.443958	0.76115	0.777757	4.87E-06
CHMP4B	32.05286	59.56277	0.893958	6.59E-18
CHMP4C	2.545459	6.03191	1.244689	2.37E-14
CHMP6	7.3808	13.82232	0.905151	7.64E-20
CHMP7	4.307537	5.458556	0.341656	0.006106
GSDMD	12.95306	31.07515	1.262469	2.96E-22
GSDME	0.421524	1.127308	1.419194	0.000548
HMGB1	11.3255	15.98758	0.497377	2.20E-09
IL18	1.569285	2.267976	0.531298	0.525646
IL1A	0.010728	0.024858	1.212383	0.007353
IL1B	0.87439	0.418351	-1.06356	2.07E-10
TP53	4.954549	8.223167	0.73094	5.80E-06
CASP6	5.030208	6.864277	0.44849	3.49E-05
CASP8	1.376885	2.831424	1.040119	1.52E-15
CASP9	1.788567	2.520602	0.494964	3.54E-05
GPX4	118.5358	188.6939	0.670725	1.28E-10
GSDMB	1.344026	3.636747	1.436088	1.66E-13
GSDMC	0.008303	0.234081	4.817161	8.32E-19
IL6	0.867032	0.320722	-1.43476	3.10E-05
NLRP1	0.40596	1.071783	1.400604	1.31E-09
NLRP3	0.351909	0.279273	-0.33353	0.002313
NLRP6	1.072124	1.192416	0.153417	0.001136
NLRP7	0.013511	0.020493	0.60107	0.002028
NOD1	0.325471	0.663693	1.027988	4.82E-14
NOD2	0.19404	0.340295	0.810434	0.007793
TNF	0.249332	0.245633	-0.02157	0.052419
