# Supplementary material for: The pyroptosis-related gene signature predicts prognosis and indicates immune activity in hepatocellular carcinoma
Source: Mol Med. 2022 Feb 5;28:16. doi: 10.1186/s10020-022-00445-0 (PMC8818170; doi:10.1186/s10020-022-00445-0)
Supplement: Supplementary file 2 — Additional file 2: Table S2. Differentially expressed genes. [file 10020_2022_445_MOESM2_ESM.pdf]

Table S2 Differentially expressed genes

gene	lowMean	highMean	logFC	pValue	fdr
CYP3A4	7.898101	6.479047	-1.41905	3.27E-05	0.000116
MCM3	5.254856	5.895022	0.640165	3.41E-11	9.01E-10
F12	9.02026	8.126904	-0.89336	2.26E-08	2.29E-07
ANXA5	7.070832	7.700991	0.630158	9.40E-09	1.09E-07
SLC6A12	5.298526	4.346406	-0.95212	1.72E-11	5.31E-10
IGSF3	3.526241	4.417293	0.891051	1.03E-16	5.98E-14
ASGR1	8.790918	8.054241	-0.73668	2.47E-14	2.93E-12
DTYMK	5.230751	5.843743	0.612992	1.55E-13	1.20E-11
LMNB1	4.729678	5.413635	0.683957	1.89E-10	3.78E-09
TFF2	2.729505	3.326458	0.596954	1.23E-06	6.89E-06
LMNB2	4.716422	5.340658	0.624237	2.74E-12	1.21E-10
SPP1	6.264279	8.071997	1.807717	9.46E-09	1.09E-07
MCM6	4.928342	5.569755	0.641414	2.09E-11	6.18E-10
MMD	4.171513	4.828597	0.657085	2.23E-11	6.45E-10
APOA4	4.435754	5.11289	0.677136	0.031594	0.04906
CDO1	8.243294	7.059843	-1.18345	3.55E-14	4.00E-12
AHSG	10.93955	10.13331	-0.80624	0.000408	0.00107
MCM4	4.897091	5.568394	0.671303	1.10E-11	3.68E-10
STK39	3.882167	4.49486	0.612693	6.29E-08	5.33E-07
HSD11B1	7.976531	7.220306	-0.75622	0.006061	0.01147
SCGN	4.310471	5.021217	0.710746	5.33E-05	0.000177
PFKFB3	4.678195	5.275785	0.59759	0.00013	0.000393
DCXR	9.482119	8.643617	-0.8385	4.95E-08	4.36E-07
HGFAC	5.870147	4.475569	-1.39458	9.23E-09	1.07E-07
ENO1	8.978525	9.656539	0.678013	9.94E-15	1.31E-12
CTH	6.589081	5.784623	-0.80446	2.34E-06	1.18E-05
GYS2	5.725454	4.85299	-0.87246	4.84E-07	3.07E-06
ECT2	3.39184	4.152289	0.760448	2.73E-15	6.28E-13
BAK1	4.442403	5.247505	0.805102	3.70E-20	1.71E-16
CXCL1	3.681217	4.558975	0.877758	2.43E-06	1.22E-05
TK1	5.633809	6.370776	0.736966	7.14E-10	1.24E-08
ALDH2	7.90239	7.28598	-0.61641	4.84E-11	1.17E-09
SULT1C2	3.68969	4.547545	0.857855	1.31E-11	4.26E-10
CCL20	5.082086	5.886347	0.80426	8.17E-05	0.000259
MMP7	3.918272	4.617131	0.69886	1.48E-05	5.87E-05
TACC3	3.931608	4.633592	0.701984	3.16E-13	2.32E-11
SULT2A1	9.172646	8.462712	-0.70993	0.001294	0.002933
ITGA3	3.402424	4.000474	0.59805	0.001838	0.003991
AGXT	9.511097	8.696304	-0.81479	4.28E-06	2.01E-05
CAV2	5.011808	5.640408	0.6286	2.49E-08	2.48E-07
RGN	7.491299	6.706324	-0.78497	4.62E-11	1.13E-09
C4BPA	9.690734	8.612333	-1.0784	2.05E-11	6.09E-10
VNN1	6.159216	6.962243	0.803027	8.63E-05	0.000271
ASRGL1	3.005094	3.610393	0.605298	1.89E-11	5.71E-10
SPAG5	4.695742	5.321309	0.625567	4.05E-11	1.02E-09
FMO3	8.34301	7.477153	-0.86586	7.51E-08	6.19E-07
PFN2	3.662308	4.428873	0.766566	3.13E-08	2.95E-07
RFC4	4.25769	4.861091	0.603401	3.64E-12	1.53E-10
ANXA13	4.001024	4.625285	0.624261	3.72E-05	0.00013
TRIP13	3.136033	3.848588	0.712555	4.67E-17	3.09E-14
BIRC5	4.434837	5.434961	1.000124	5.42E-15	1.05E-12
TAT	8.118161	6.969022	-1.14914	1.50E-05	5.94E-05
UCHL1	2.895269	3.499432	0.604163	0.000144	0.000429
RAMP3	5.336945	4.605007	-0.73194	9.77E-11	2.11E-09
ITGB4	3.510126	4.152741	0.642615	1.26E-06	6.98E-06

PSPH	4.996092	5.688212	0.69212	8.70E-13	4.85E-11
HPD	9.560179	8.091026	-1.46915	5.21E-08	4.57E-07
CYP4A11	7.255642	6.423642	-0.832	1.06E-06	6.03E-06
SPARCL1	6.632629	5.853102	-0.77953	2.03E-07	1.43E-06
UBE2S	3.944669	4.635261	0.690593	4.74E-14	4.99E-12
KIF2C	3.473943	4.331325	0.857382	4.70E-16	1.39E-13
ENAH	4.722589	5.445111	0.722523	4.82E-16	1.39E-13
G6PD	4.583884	5.639289	1.055405	2.85E-15	6.28E-13
CCNB2	3.595091	4.344521	0.74943	2.03E-12	9.48E-11
CYP2A6	7.422023	6.687228	-0.73479	0.022563	0.036252
CDC20	4.380094	5.477988	1.097894	6.75E-15	1.15E-12
MAT1A	9.35846	8.647914	-0.71055	8.59E-09	1.00E-07
MCM7	6.187237	6.774533	0.587296	7.36E-12	2.79E-10
DAB2	4.579421	5.196902	0.617481	4.46E-08	3.99E-07
HPX	10.50133	9.686139	-0.81519	3.89E-07	2.54E-06
DDR1	3.981023	4.757963	0.77694	4.02E-08	3.63E-07
CXCL5	2.708286	3.347299	0.639013	5.68E-07	3.51E-06
STX3	4.28685	4.921501	0.634651	4.93E-14	5.07E-12
CTHRC1	3.73822	4.341985	0.603765	0.000259	0.000715
SNX7	4.565593	5.301087	0.735494	7.80E-15	1.17E-12
GNPDA1	4.707114	5.305765	0.598651	3.12E-15	6.56E-13
FGA	12.0841	11.44863	-0.63546	4.67E-06	2.16E-05
GAL3ST1	3.704832	4.567013	0.86218	3.19E-06	1.56E-05
TUSC3	3.744963	4.343703	0.59874	5.15E-05	0.000172
SPP2	7.223815	6.284262	-0.93955	1.30E-05	5.26E-05
ZWINT	4.451136	5.286065	0.834929	1.25E-13	1.02E-11
SLC16A3	3.359807	4.09976	0.739953	3.28E-09	4.48E-08
PDK4	6.444105	5.777766	-0.66634	1.17E-05	4.82E-05
CES2	7.46722	6.829752	-0.63747	3.99E-05	0.000138
CDC6	3.468464	4.271055	0.802591	3.19E-16	1.39E-13
ALDOA	6.849151	7.653742	0.804592	2.20E-12	1.00E-10
CD24	5.461171	6.951523	1.490352	8.08E-12	2.97E-10
LAPTM4B	5.959517	6.929506	0.969989	3.53E-13	2.56E-11
HP	11.06564	10.10617	-0.95946	1.02E-06	5.84E-06
MPZL1	5.070513	5.689433	0.618919	9.79E-12	3.41E-10
CYP2C9	7.639093	6.508837	-1.13026	1.06E-09	1.71E-08
AR	5.592091	4.976173	-0.61592	2.32E-07	1.63E-06
FGFR2	4.375662	5.004014	0.628352	0.000226	0.000635
UBE2C	4.890763	5.92169	1.030926	1.89E-12	9.01E-11
CYP1A1	4.989932	3.897776	-1.09216	1.84E-05	7.10E-05
TPX2	4.841065	5.689849	0.848784	8.71E-14	8.22E-12
CD14	8.996597	8.323717	-0.67288	7.17E-08	5.96E-07
CPB2	9.607876	8.909594	-0.69828	1.15E-08	1.28E-07
PFKFB1	5.143093	4.510501	-0.63259	3.24E-05	0.000115
ITPR3	2.749459	3.352097	0.602638	3.38E-06	1.64E-05
SFN	4.507511	5.75267	1.245159	6.65E-10	1.16E-08
CBX1	4.827893	5.434018	0.606125	8.31E-14	8.01E-12
FEN1	4.780034	5.406358	0.626325	1.06E-12	5.79E-11
ATP1B3	4.963415	5.697423	0.734008	2.46E-11	6.94E-10
CDKN3	4.443211	5.171183	0.727972	1.26E-09	1.95E-08
CCNB1	4.468916	5.416171	0.947256	3.55E-15	7.15E-13
ANXA2	6.284549	6.892156	0.607607	2.01E-08	2.08E-07
CDC25B	4.8361	5.484379	0.648279	4.78E-11	1.16E-09
KDELR3	5.032171	5.686836	0.654665	2.54E-07	1.75E-06
MT1A	6.227559	5.495258	-0.7323	0.00014	0.000418
BUB1B	3.405361	4.086948	0.681587	1.46E-14	1.83E-12
DUSP9	4.077361	4.710696	0.633335	0.001326	0.002996
SPINK1	6.723951	7.561385	0.837434	0.016406	0.027536

PON3	7.099786	6.463186	-0.6366	7.56E-09	9.04E-08
PYGB	5.40719	6.044371	0.63718	4.71E-13	2.99E-11
NUSAP1	4.935891	5.680905	0.745014	9.12E-12	3.25E-10
C7	5.65151	4.820255	-0.83126	0.000124	0.000377
NDRG1	6.039881	6.769635	0.729754	6.11E-09	7.48E-08
CFHR4	5.674184	4.496646	-1.17754	4.59E-12	1.86E-10
TSPAN13	5.266025	5.854075	0.58805	0.000198	0.000567
MYBL2	4.020524	5.347165	1.326641	6.43E-18	9.92E-15
GSDME	3.161541	3.833841	0.6723	4.70E-18	9.92E-15
GLS	4.603038	5.202601	0.599563	2.06E-10	4.08E-09
C4BPB	8.275137	7.602226	-0.67291	2.45E-08	2.45E-07
GMNN	5.763621	6.361547	0.597925	3.22E-09	4.42E-08
STMN1	5.027294	5.768931	0.741637	3.38E-11	9.00E-10
CCL21	5.741392	5.08786	-0.65353	0.005838	0.011094
CYP2B6	6.267821	5.64961	-0.61821	0.000847	0.002026
PAFAH1B3	4.934941	5.830991	0.896049	1.17E-13	1.02E-11
F9	7.438108	6.684527	-0.75358	0.000566	0.001421
CENPF	3.446956	4.097488	0.650532	1.10E-11	3.68E-10
SLC2A2	7.386903	6.650042	-0.73686	4.40E-07	2.83E-06
AOX1	8.400158	7.299841	-1.10032	8.13E-09	9.60E-08
HRG	9.482599	8.842989	-0.63961	0.003603	0.007243
CAPG	5.319884	6.088571	0.768687	1.95E-08	2.02E-07
BHMT	7.388463	6.771195	-0.61727	0.007634	0.01405
CLSTN1	5.173701	5.773857	0.600156	2.64E-08	2.60E-07
PLK1	3.487929	4.248079	0.76015	7.39E-16	1.90E-13
SLC13A3	4.622106	3.844328	-0.77778	0.000315	0.000851
DPYS	7.786216	7.180342	-0.60587	0.002385	0.005015
ITGA5	5.654437	6.254503	0.600067	3.48E-11	9.15E-10
CDK4	5.302999	5.954203	0.651204	1.72E-11	5.31E-10
MT1X	7.472694	6.683977	-0.78872	0.000558	0.001401
SERPINF2	9.970557	9.201232	-0.76932	3.67E-09	4.89E-08
ANG	9.583809	8.800132	-0.78368	1.50E-08	1.60E-07
PI3	3.092219	3.713734	0.621515	5.33E-06	2.43E-05
PTTG1	5.053325	5.905619	0.852294	3.27E-11	8.85E-10
S100P	4.402581	5.236342	0.833761	0.003341	0.006772
GINS1	3.517712	4.274154	0.756442	2.81E-17	2.45E-14
HLF	5.471317	4.868623	-0.60269	1.58E-05	6.20E-05
NCAPH	3.446529	4.275926	0.829397	4.78E-16	1.39E-13
HMGA1	6.604243	7.418596	0.814353	6.12E-13	3.73E-11
EHHADH	7.449395	6.857477	-0.59192	1.64E-05	6.43E-05
DSG2	4.203828	5.290764	1.086936	3.18E-17	2.45E-14
FABP4	4.763634	4.030658	-0.73298	1.38E-07	1.03E-06
CKAP4	6.573889	7.210607	0.636718	1.59E-11	5.09E-10
ADH1B	9.178583	7.622207	-1.55638	7.16E-13	4.20E-11
KNG1	10.36019	9.72718	-0.63301	0.000111	0.000341
GPT	6.68714	6.040624	-0.64652	5.41E-06	2.46E-05
TUBG1	5.996407	6.593634	0.597227	7.20E-15	1.15E-12
HSD17B6	8.613323	7.898219	-0.7151	1.59E-05	6.25E-05
NEK2	4.076236	4.790773	0.714536	7.41E-12	2.79E-10
GTSE1	3.258536	3.850231	0.591695	2.89E-14	3.34E-12
SLPI	7.624706	8.381991	0.757285	0.002534	0.005272
RENBP	4.58426	5.20227	0.61801	3.15E-05	0.000113
SLC6A8	3.729231	4.439077	0.709846	4.91E-05	0.000165
CYP2E1	8.662829	6.86001	-1.80282	3.38E-09	4.61E-08
SLC10A1	6.946893	5.81641	-1.13048	3.94E-06	1.86E-05
E2F1	4.395363	5.26526	0.869898	5.98E-12	2.32E-10
CCNE1	3.258488	3.846631	0.588144	4.96E-12	1.99E-10
SMOX	4.057154	4.665245	0.608091	4.00E-08	3.62E-07

SOX4	4.142818	5.061892	0.919074	5.67E-11	1.32E-09
FOXM1	3.712329	4.537431	0.825102	5.35E-13	3.30E-11
NDC80	3.51316	4.113718	0.600558	4.36E-12	1.79E-10
TYMS	5.061131	5.740306	0.679176	1.15E-09	1.85E-08
KCNJ8	6.037733	5.329756	-0.70798	8.43E-09	9.91E-08
SRC	4.088385	4.675121	0.586737	2.04E-08	2.11E-07
NR1I3	6.307699	5.583269	-0.72443	1.25E-05	5.07E-05
IMPDH1	3.723031	4.323309	0.600278	1.38E-07	1.03E-06
HOMER3	4.373171	4.978539	0.605368	1.57E-07	1.16E-06
CYP1A2	4.9654	3.899686	-1.06571	0.000156	0.000459
CD5L	4.312734	3.489475	-0.82326	2.56E-09	3.66E-08
AQP9	8.531752	7.0717	-1.46005	5.40E-11	1.28E-09
MKI67	3.268921	3.935269	0.666348	1.69E-11	5.31E-10
LCAT	6.012126	5.290978	-0.72115	2.45E-08	2.45E-07
GPC3	7.794902	8.529491	0.734589	0.005449	0.010406
PLG	9.175379	8.303222	-0.87216	2.10E-07	1.48E-06
KPNA2	5.643015	6.450251	0.807236	4.47E-16	1.39E-13
APOM	8.702621	8.080337	-0.62228	9.39E-05	0.000293
TMSB10	10.11199	10.77658	0.664586	4.63E-06	2.14E-05
CYP27A1	9.3028	8.530647	-0.77215	2.23E-11	6.45E-10
CLDN4	3.527986	4.503669	0.975683	6.02E-07	3.70E-06
KRT19	3.330862	3.979436	0.648573	0.008764	0.015915
PRG4	5.877511	6.526551	0.64904	0.000584	0.001459
IGF2	6.891293	6.222643	-0.66865	0.019926	0.032673
MT2A	9.401612	8.687717	-0.7139	0.000873	0.00208
ECM2	5.329197	4.744144	-0.58505	9.23E-09	1.07E-07
ALDOB	11.05539	10.41598	-0.6394	0.006194	0.011694
ADH1A	8.722421	7.798044	-0.92438	5.68E-08	4.88E-07
AZGP1	9.607453	8.87484	-0.73261	2.87E-06	1.41E-05
EZH2	3.694363	4.359732	0.665369	7.08E-15	1.15E-12
SLC22A1	7.029178	6.111476	-0.9177	0.000456	0.001179
S100A9	5.193497	5.848266	0.654769	0.001857	0.004029
BDH1	6.622462	6.00012	-0.62234	8.08E-08	6.57E-07
NKG7	5.292607	4.660338	-0.63227	5.54E-09	6.88E-08
TRIB3	5.454035	6.127396	0.673361	1.85E-10	3.72E-09
ALDH1L1	7.040199	6.354721	-0.68548	0.000454	0.001176
PITX1	2.616034	3.307195	0.691161	3.73E-09	4.96E-08
PON1	8.27438	7.479634	-0.79475	4.64E-07	2.96E-06
DBN1	4.447031	5.162071	0.715039	1.73E-07	1.25E-06
C6	7.32287	6.518602	-0.80427	1.36E-06	7.46E-06
S100A11	7.777318	8.506732	0.729414	3.40E-05	0.00012
PRAME	2.809918	3.411408	0.601491	0.000208	0.000591
IGFBP2	7.884542	7.224417	-0.66012	0.000823	0.00197
PKMYT1	3.136729	3.730989	0.59426	1.04E-13	9.44E-12
FMO5	7.348523	6.719238	-0.62929	7.17E-07	4.28E-06
TROAP	3.82138	4.523848	0.702469	1.81E-12	8.71E-11
CLGN	3.680996	4.581656	0.90066	5.52E-11	1.30E-09
MBL2	6.479818	5.744268	-0.73555	1.64E-05	6.42E-05
PCK1	7.88365	6.754452	-1.1292	6.32E-07	3.85E-06
MELK	3.500431	4.253866	0.753434	4.32E-14	4.65E-12
SLC1A2	4.569845	3.881984	-0.68786	2.18E-06	1.11E-05
DNASE1L3	4.24669	3.393467	-0.85322	3.73E-13	2.61E-11
APOC1	12.50797	11.89464	-0.61333	6.32E-06	2.82E-05
CENPA	3.083986	3.775238	0.691253	3.33E-16	1.39E-13
SLC17A2	5.983151	5.378896	-0.60426	4.78E-05	0.000162
VEGFB	5.959443	6.757713	0.79827	6.34E-09	7.70E-08
OTC	7.02573	6.201534	-0.8242	7.92E-07	4.62E-06
ABCC4	3.351745	4.000505	0.64876	1.24E-13	1.02E-11

ITIH3	8.957706	8.219234	-0.73847	9.25E-08	7.35E-07
BACE2	3.676718	4.268065	0.591348	0.000866	0.002068
RAP1GAP	4.289462	4.944433	0.654971	5.36E-07	3.34E-06
IGFBP1	9.511334	8.803218	-0.70812	6.34E-05	0.000207
ITIH1	9.774077	9.172227	-0.60185	1.15E-07	8.73E-07
MCM2	4.427816	5.40533	0.977514	7.08E-15	1.15E-12
SEC14L2	6.352431	5.753588	-0.59884	7.18E-05	0.000231
SERPINC1	11.56543	10.46856	-1.09687	3.13E-08	2.95E-07
LGALS4	7.06131	7.923185	0.861875	9.55E-05	0.000297
C8A	7.075733	6.28775	-0.78798	1.26E-07	9.46E-07
SHBG	5.690763	5.098362	-0.5924	0.000736	0.001788
RRM2	4.552833	5.387349	0.834516	8.50E-13	4.80E-11
CPS1	8.477988	7.508966	-0.96902	0.000192	0.000554
CA9	3.007897	4.026652	1.018756	2.62E-09	3.74E-08
TMEM106	5.687408	6.387956	0.700548	1.26E-13	1.02E-11
